# Profiling births via Caesarean delivery in Northern Ireland (2011-2019) using the Robson classification: a population-based cohort study using administrative maternity system data.

**DOI:** 10.23889/ijpds.v9i5.2740

**Published:** 2024-09-18

**Authors:** Orla McBride, Enya Redican, Jamie Murphy

**Affiliations:** 1 Ulster University

## Objective and Approach

Caesarean delivery (CD) rates are increasing globally. Over one-third of babies born in Northern Ireland (NI) during 2021-22 were via CD. Reducing the CD rate was cited as an important public health priority in the Strategy for Maternity Care in Northern Ireland (2012-2018). In 2015, WHO advocated that the Robson Ten Group Classification System (TGCS) be used to audit, analyse, and compare CD rates across countries. TGCS is currently not implemented in NI.

## Results

Analysis of NI’s administrative maternity system (NIMATS) is on-going (accessed via Honest Broker Service Project P045) to address this study’s objectives, which will be completed for presentation: (1) describe the characteristics (maternal, obstetric practice, and fetal/infant features) of all deliveries during 2011-2019; (2) group all births over this period into TGCS categories and calculate the relative size of each group and the absolute contribution of each group to the overall CD rate; (3) compare the NI population distribution of TGCS group-specific CD rates against WHO-recommended benchmarks; (4) test for temporal trends in CD rates here over time; and (5) estimate the ability of a range of maternal, infant, and obstetric factors in predicting the overall CD rate and by TGCS group in NI over time.

## Conclusions

Application of the Robson TGCS to NIMATS data has potential to address a critical knowledge gap relating to the use of CD in NI.

## Implications

Avenues to extend this work to investigate longer-term outcomes (via possible linkage to the NI Child Health System) will be considered.

